# Comparative analysis of response to selection with three insecticides in the dengue mosquito *Aedes aegypti* using mRNA sequencing

**DOI:** 10.1186/1471-2164-15-174

**Published:** 2014-03-05

**Authors:** Jean-Philippe David, Frédéric Faucon, Alexia Chandor-Proust, Rodolphe Poupardin, Muhammad Asam Riaz, Aurélie Bonin, Vincent Navratil, Stéphane Reynaud

**Affiliations:** 1 Laboratoire d’Ecologie Alpine (LECA), UMR CNRS 5553, Grenoble, France; 2 Université Grenoble Alpes, Grenoble, France; 3 Environmental and Systems Biology (BEeSy), Université Grenoble Alpes, Grenoble, France; 4 Vector Biology group, Liverpool School of Tropical Medicine, Liverpool, UK; 5 Department of Entomology, University of Sargodha, Sargodha, Pakistan; 6 Pôle Rhône Alpes de Bioinformatique, Université Lyon 1, Villeurbanne, France

**Keywords:** RNA sequencing, RNA-seq, Insecticide resistance, Mosquito, Dengue, Detoxification enzymes, Cytochrome P450 monooxygenase, CYP, Cuticle, Transporters

## Abstract

**Background:**

Mosquito control programmes using chemical insecticides are increasingly threatened by the development of resistance. Such resistance can be the consequence of changes in proteins targeted by insecticides (target site mediated resistance), increased insecticide biodegradation (metabolic resistance), altered transport, sequestration or other mechanisms. As opposed to target site resistance, other mechanisms are far from being fully understood. Indeed, insecticide selection often affects a large number of genes and various biological processes can hypothetically confer resistance. In this context, the aim of the present study was to use RNA sequencing (RNA-seq) for comparing transcription level and polymorphism variations associated with adaptation to chemical insecticides in the mosquito *Aedes aegypti*. Biological materials consisted of a parental susceptible strain together with three child strains selected across multiple generations with three insecticides from different classes: the pyrethroid permethrin, the neonicotinoid imidacloprid and the carbamate propoxur.

**Results:**

After ten generations, insecticide-selected strains showed elevated resistance levels to the insecticides used for selection. RNA-seq data allowed detecting over 13,000 transcripts, of which 413 were differentially transcribed in insecticide-selected strains as compared to the susceptible strain. Among them, a significant enrichment of transcripts encoding cuticle proteins, transporters and enzymes was observed. Polymorphism analysis revealed over 2500 SNPs showing > 50% allele frequency variations in insecticide-selected strains as compared to the susceptible strain, affecting over 1000 transcripts. Comparing gene transcription and polymorphism patterns revealed marked differences among strains. While imidacloprid selection was linked to the over transcription of many genes, permethrin selection was rather linked to polymorphism variations. Focusing on detoxification enzymes revealed that permethrin selection strongly affected the polymorphism of several transcripts encoding cytochrome P450 monooxygenases likely involved in insecticide biodegradation.

**Conclusions:**

The present study confirmed the power of RNA-seq for identifying concomitantly quantitative and qualitative transcriptome changes associated with insecticide resistance in mosquitoes. Our results suggest that transcriptome modifications can be selected rapidly by insecticides and affect multiple biological functions. Previously neglected by molecular screenings, polymorphism variations of detoxification enzymes may play an important role in the adaptive response of mosquitoes to insecticides.

## Background

Mosquitoes are vectors of several human diseases representing a major burden for public health worldwide [1]. Half of the world’s population is exposed to malaria while dengue fever represents a burden in over 100 countries with 2.5 billion people at risk [2,3]. Since the 1950s, chemical insecticides have been massively used for controlling mosquito populations but their efficacy is now threatened by resistance mechanisms developed by insects. In absence of efficient alternatives, characterizing molecular mechanisms underlying resistance is a key step for improving resistance management strategies.

Resistance to insecticides can be the consequence of different mechanisms, such as a mutation of the proteins targeted by the insecticide (target-site insensitivity), a lower penetration of the insecticide, its sequestration, or its biodegradation (metabolic resistance) [4,5]. Target-site insensitivity and metabolic resistance are known as the two main resistance mechanisms in mosquitoes [4,6]. Mutations causing target-site insensitivity are well-characterized in mosquitoes and molecular tests detecting these mutations are available for species of public health importance [7-12]. Metabolic resistance has been reported worldwide and usually involves detoxification enzymes such as cytochrome P450 monooxygenases (P450s or CYPs for genes), carboxy/cholinesterases (CCEs), glutathione S-transferases (GSTs) and UDP glucosyl-transferases (UGTs) [4,6,13,14]. However, due to the large number of mosquito genes encoding detoxification enzymes [15-17] pinpointing those responsible for resistance remains challenging [18]. Metabolic resistance has been mostly associated with an increased level of detoxification enzymes in resistant populations and multiple candidate genes have been identified by microarray screenings [6,18-21]. In contrast, polymorphism variations potentially affecting the functionality of detoxification enzymes have been hardly studied in mosquitoes despite evidences suggesting that this phenomenon may play a role in insecticide resistance [14,22]. Recently, polymorphism of a P450 gene has been associated with pyrethroid resistance in the mosquito *culex pipiens*[23] and a reduction of sequence diversity in two P450 genes conferring resistance to pyrethroids has been observed in *Anopheles funestus*[24]. This suggests that a deep analysis of the polymorphism associated with resistance can improve our understanding of mechanisms developed by mosquitoes to resist insecticides. Today, such knowledge gap can be overcome by high throughput sequencing approaches such as mRNA sequencing (RNA-seq), which can generates concomitantly gene expression and polymorphism data over the whole transcriptome from a single experiment [25-27].

In this context, the aim of the present study was to use RNA-seq for investigating transcription level and polymorphism variations associated with adaptation to three insecticides from distinct chemical families in the mosquito *Aedes aegypti*. A susceptible strain was selected with the pyrethroid permethrin, the neonicotinoid imidacloprid or the carbamate propoxur, to produce three resistant strains. After ten generations of selection, the constitutive resistance level of each resistant strain was measured and the transcriptome of each strain was deep sequenced. After mapping cDNA reads to the genome, gene expression and polymorphism variations linked to insecticide selection were identified and compared across strains. Results are discussed in regards of known and new putative adaptive mechanisms conferring insecticide resistance in mosquitoes.

## Results

### Insecticide resistance levels

After ten generations of larval selection with the insecticides permethrin (Perm-R strain), imidacloprid (Imida-R strain) or propoxur (Propo-R strain), bioassays revealed a constitutive increased resistance of each selected strain to its respective insecticide as compared to the parental susceptible strain (Table 1). Resistance levels of the Perm-R and Imida-R strains were moderate but significant (3.78 fold and 5.85 fold respectively). Although significant, the resistance level of the Propo-R strain to propoxur was considerably lower (1.84 fold).

**Table 1:** Resistance levels after insecticide selection

**Insecticide**	**Strain**	**LC**_**50 **_**(μg/L)**	**CI**_**95% **_**(μg/L)**	**RR**_**50**_	**CI**_**95%**_
Permethrin	Susceptible	2.52	2.29 - 2.77	-	-
	Perm-R	9.47	6.21 - 14.43	**3.78**	2.24 - 6.30
Imidacloprid	Susceptible	240.30	208.74 - 276.67	-	-
	Imida-R	1406.40	1236.24 - 1599.87	**5.85**	4.47 - 7.66
Propoxur	Susceptible	441.20	392.16 - 496.34	-	-
	Propo-R	813.60	743.47 - 890.23	**1.84**	1.50 - 2.27

Resistance ratios were computed from LC_50_ values as compared to the susceptible parental strain.

Resistance ratios with confidence intervals not overlapping the value of 1 are shown in bold.

### Sequencing, read mapping and genome re-annotation

More than 269 million 75 bp cDNA reads were sequenced across all samples (Additional file 1: Table S1). Each mosquito strain was represented by two cDNA libraries with an average of 33.6 million reads per library. More than 80% of reads were successfully mapped on the *Ae. aegypti* genome. Filtering on sequence quality and mapping score retained more than 174 million reads (~ 65%). For each strain, RPKMs (Reads Per Kilobase of exon model per Million reads) obtained from the two cDNA library replicates were well correlated (Additional file 2: Figure S1), indicating moderate variations between technical replicates. In consequence, reads from technical replicates were pooled for further analyses.

A transcription signal was detected for 85% of known *Ae. aegypti* genes. Comparing transcript coverage between strains revealed similar distributions with RPKMs spanning more than 6 Logs and a median transcript coverage of 4.4 RPKM (~300 reads/Kb) (Additional file 3: Figure S2). Comparing genome annotation with the distribution and coverage of reads suggested incorrect gene boundary annotations for more than 3000 transcripts and identified more than 500 isolated novel transcription events (NTEs) based on their transcription signal, structure and high distance to known transcripts (Additional file 4: Table S2 and Additional file 5: Table S3). Distribution of mapped reads across the whole *Ae. aegypti* genome can be accessed at http://vectorbase.org using the *‘configure this page’* and *‘RNAseq alignments’* options of the genome browser (tracks ‘Bora-Bora control’, ‘Perm-R’, ‘Imida-R’ and ‘Propo-R’).

### Differential transcription in insecticide-selected strains

Differential transcription analysis was performed on the 13105 transcripts showing a transcription signal higher than 0.5 RPKM in all strains. A total of 463 transcripts (~ 3.5%) including 413 known transcripts and 50 NTEs were considered differentially transcribed in any insecticide-selected strain as compared to the susceptible strain (> 3 fold in either direction and adjusted P value < 10^-15^; Table 2). Such threshold appeared biologically relevant as less than 2% of transcription ratios belonging to 140 housekeeping genes (all ribosomal proteins, actins and tubulins) were found differentially transcribed as compared to the parental strain. Cross-comparison of transcription ratios (TRs) obtained from RNA-seq and DNA microarray was performed for the Imida-R strain from the same biological samples (Additional file 6: Figure S3). This comparison revealed a good correlation between the two techniques (r^2^ = 0.86, slope ~ 1, most variations < 2 fold).

**Table 2:** Differential transcription analysis overview

	**Perm-R**		**Imida-R**		**Propo-R**		**Any strains**	
	**Transcripts**	**%**	**Transcripts**	**%**	**Transcripts**	**%**	**Transcripts**	**%**
AC test P value	2858	21.8	2942	22.5	1881	14.4	4188	32.0
AC test P value and FC >3	181	1.4	339	2.6	131	1.0	463	3.5
Over transcribed	34	0.3	259	2.0	38	0.3	279	2.1
Known transcripts	34	0.3	235	1.8	37	0.3	255	1.9
New putative transcripts	0	0.0	24	0.2	1	0.0	24	0.2
Under transcribed	147	1.1	80	0.6	93	0.7	239	1.8
Known transcripts	131	1.0	70	0.5	76	0.6	210	1.6
New putative transcripts	16	0.1	10	0.1	17	0.1	29	0.2

**Figure 1:**
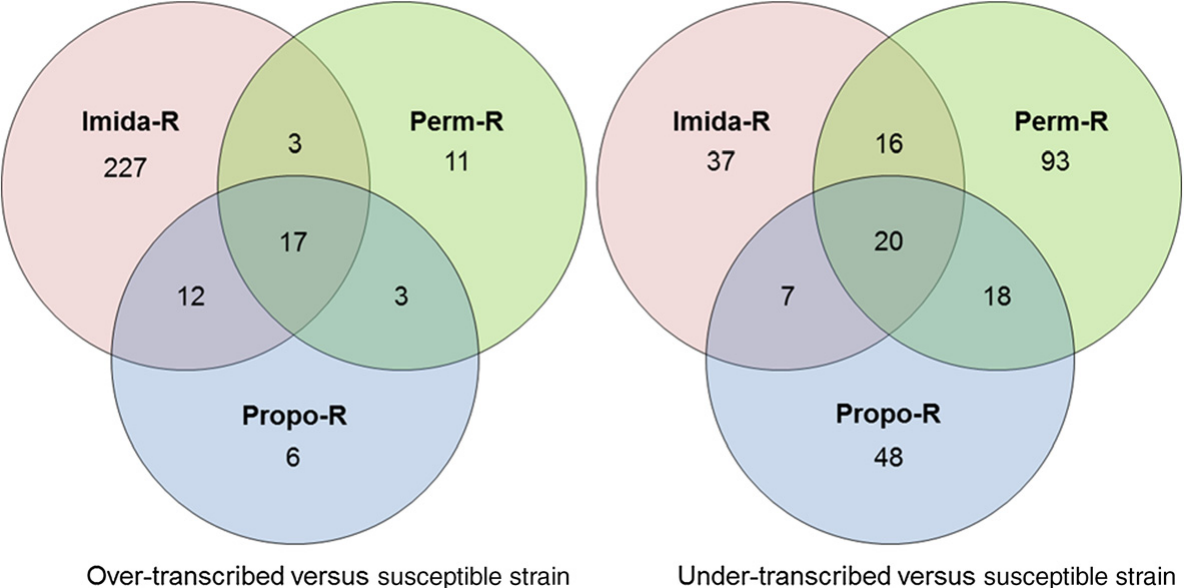
**Transcripts differentially expressed after insecticide selection.** For each Venn diagram section, the numbers of transcripts differentially expressed in any insecticide-selected strain as compared to the susceptible strain are indicated.

The balance between over- and under transcription was contrasted between each strain (Figure 1). The Imida-R strain showed the widest response to insecticide selection with 227 transcripts specifically over transcribed as compared to the susceptible strain. In contrast, fewer transcripts were affected in Perm-R and Propo-R strains with the majority of them being under transcribed. A total of 96 transcripts were found differentially expressed in multiple strains including 17 and 20 transcripts over- and under transcribed in all strains respectively.

**Figure 2:**
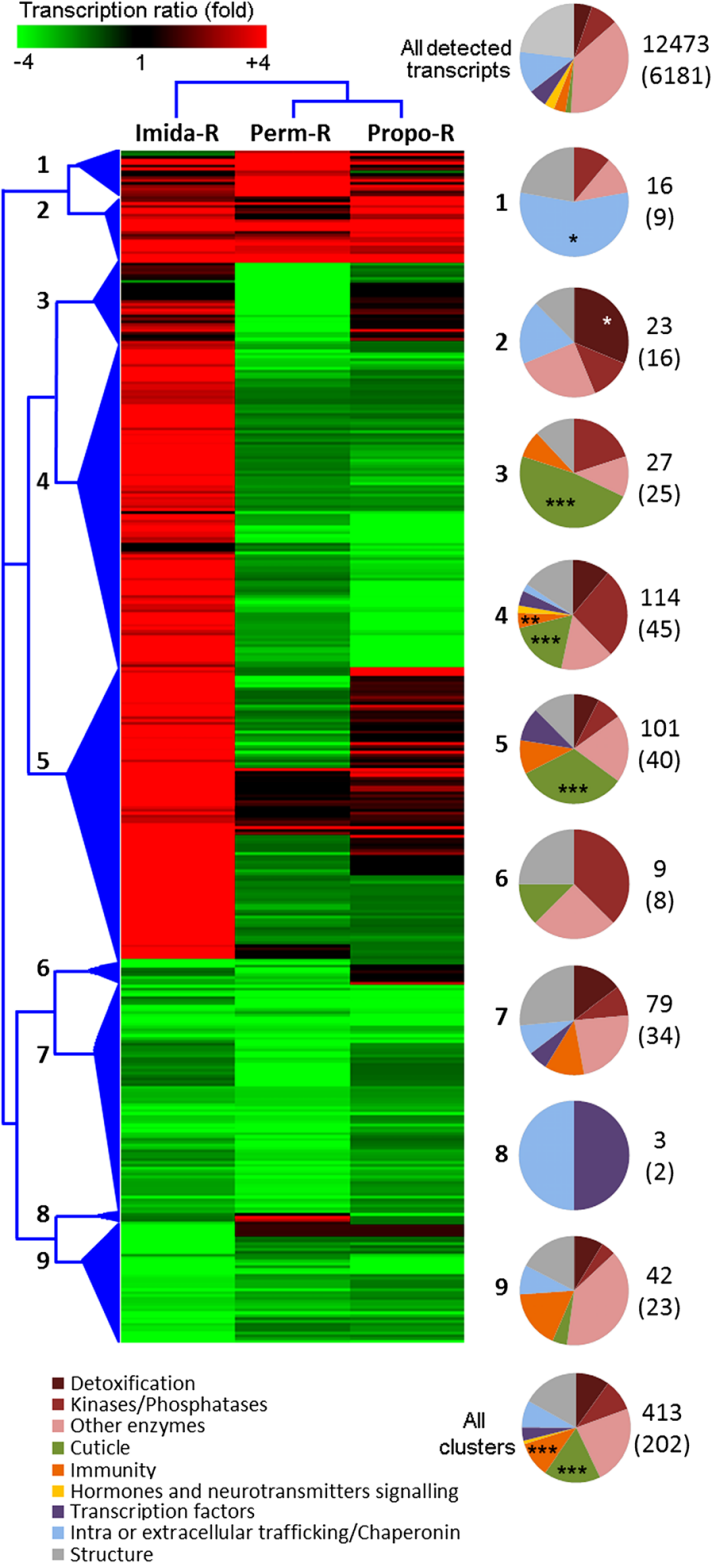
**Clustering of transcripts differentially expressed across strains.** The analysis was performed on the 413 known transcripts significantly differentially expressed in any insecticide-selected strain compared to the susceptible strain. Clustering was based on Euclidean distance of fold changes as compared to the susceptible strain and complete linkage algorithm. Pie charts describe biological functions affected within main clusters based on the number of transcripts assigned to each function. Stars indicate biological functions significantly enriched compared to their representation among all detected transcripts (Fisher’s test adjusted P value: * ≤ 0.05, ** ≤ 0.01, *** ≤ 0.001). The total number of transcripts constituting each cluster is indicated. The number of transcripts with predicted functions used for building each pie chart is shown within brackets.

A clustering analysis based on TRs as compared to the susceptible strain was performed on the 413 known transcripts differentially transcribed in any insecticide-selected strain (Figure 2 and Additional file 7: Table S4). This analysis confirmed the specific over transcription of several genes in the Imida-R strain and identified nine main transcript clusters based on their expression profile across strains. Assigning known transcripts to biological categories and comparing their frequency to all detected transcripts revealed protein families or biological functions enriched in the different clusters (Figure 2). When considering all clusters as a whole, a significant enrichment of transcripts related to immunity and cuticular proteins was observed. Cluster 1, representing transcripts strongly over transcribed in Perm-R strain included 5 hexamerins associated to cellular trafficking. These hexamerins showed very high TRs in the Perm-R strain (from 10 to 150 fold) but were also over transcribed in other insecticide-selected strains (up to 12 fold). Cluster 2, representing transcripts over transcribed in all insecticide-selected strains, showed a significant enrichment in detoxification enzymes. Cluster 3, representing transcripts strongly under transcribed in the Perm-R strain but not in others, was significantly enriched in cuticle proteins. Clusters 4 and 5 were characterized by transcripts showing a strong over transcription in the Imida-R strain and were significantly enriched in cuticular proteins. A significant enrichment in proteins potentially involved in immunity was also observed for cluster 4.

### Polymorphism variations in insecticide-selected strains

**Figure 3:**
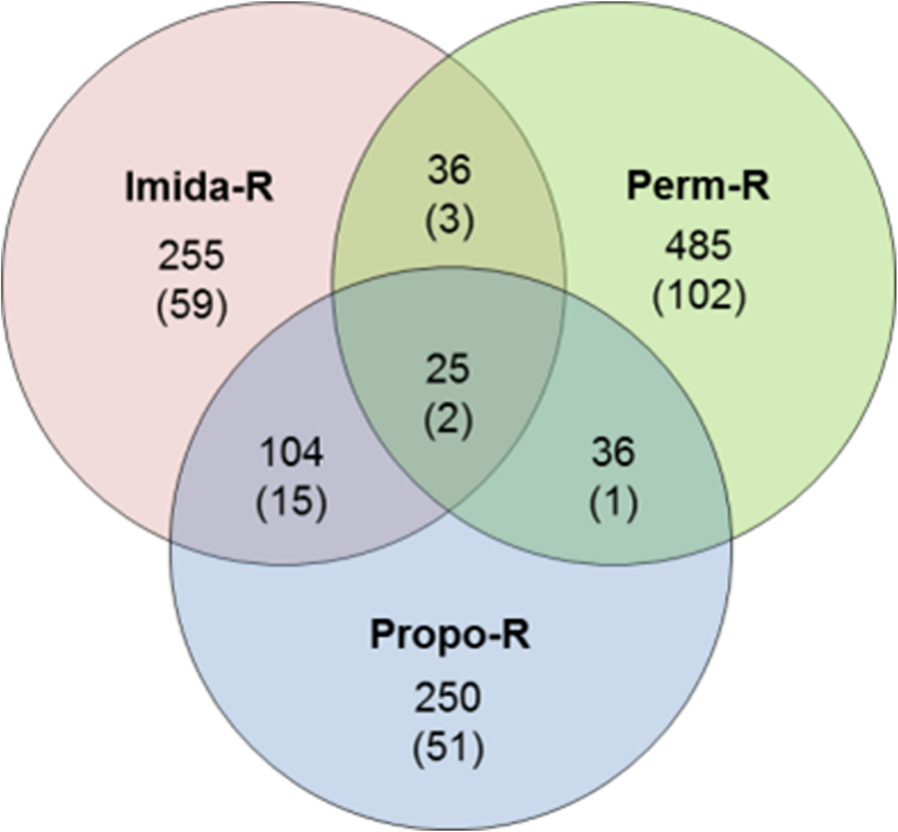
**Transcripts affected by differential SNPs.** For each Venn diagram section, the number of transcripts affected by differential SNPs is shown. The number of transcripts affected by differential SNPs predicted as non-synonymous according to genome annotation are shown within brackets.

A total of 220,499 SNP loci were identified between any strain and the *Ae. aegypti* reference genome. As expected in populations under selection and experiencing recurrent bottlenecks, a notable lower variability in insecticide-selected strains was observed as compared to the parental susceptible strain (Table 3). Comparative polymorphism analysis identified several alleles differentially represented between insecticide-selected strains and the parental susceptible strain (> 50% allele frequency difference, referred to as ‘differential SNPs’). The Perm-R strain showed more differential SNPs (1315) as compared to other insecticide-selected strains (811 and 812 for the Imida-R and Propo-R strains, respectively). Predicted genic effects of these variations were equally distributed among insecticide-selected strains with ~ 2.5% of them located in 5′UTRs, ~ 9% in 3′UTRs, ~ 60% within transcript coding sequences, ~ 5% in introns and ~ 20% within 1 kb of gene boundaries (referred to as ‘intergenic’). No significant correlation was observed between transcripts differentially transcribed and the presence of differential SNPs in their 5′ or 3′ UTRs (not shown). More than 1080 transcripts were affected by differential SNPs in at least one insecticide-selected strain (Figure 3). Up to 22 differential SNPs per transcript were observed, with 21 transcripts affected by > 10 differential SNPs. The Perm-R strain showed a higher number of transcripts affected by differential SNPs as compared to other strains (582 versus 415 and 420). Only 25 transcripts were affected by differential SNPs in all insecticide-selected strains. No differential SNP was found in transcripts encoding known insecticide target proteins such as the para-type voltage-gated sodium channel (permethrin), the acetylcholinesterase (propoxur) or the nicotinic acetylcholine receptor (imidacloprid).

**Table 3:** Differential polymorphism analysis overview

	**Perm-R**		**Imida-R**		**Propo-R**	
	**SNPs**	**%**	**SNPs**	**%**	**SNPs**	**%**
Detected SNPs	143737	100	143645	100	133892	100
Differential SNPs	1315	0.9	811	0.6	812	0.6
Genic consequences*	1403	100	881	100	908	100
5′ UTR	32	2.3	28	3.2	25	2.8
Synonymous coding	715	51.0	442	50.2	453	49.9
Non-synonymous Coding	137	9.8	95	10.8	108	11.9
Intronic	66	4.7	45	5.1	54	5.9
3′ UTR	121	8.6	88	10.0	87	9.6
Intergenic	325	23.2	184	20.9	181	19.9

*Consequences of allelic changes are only indicated for differential SNPs (*i.e.* alleles showing > 50% allelic frequency difference in any selected strain as compared to the susceptible strain). These consequences were computed based on genome annotation.

**Figure 4:**
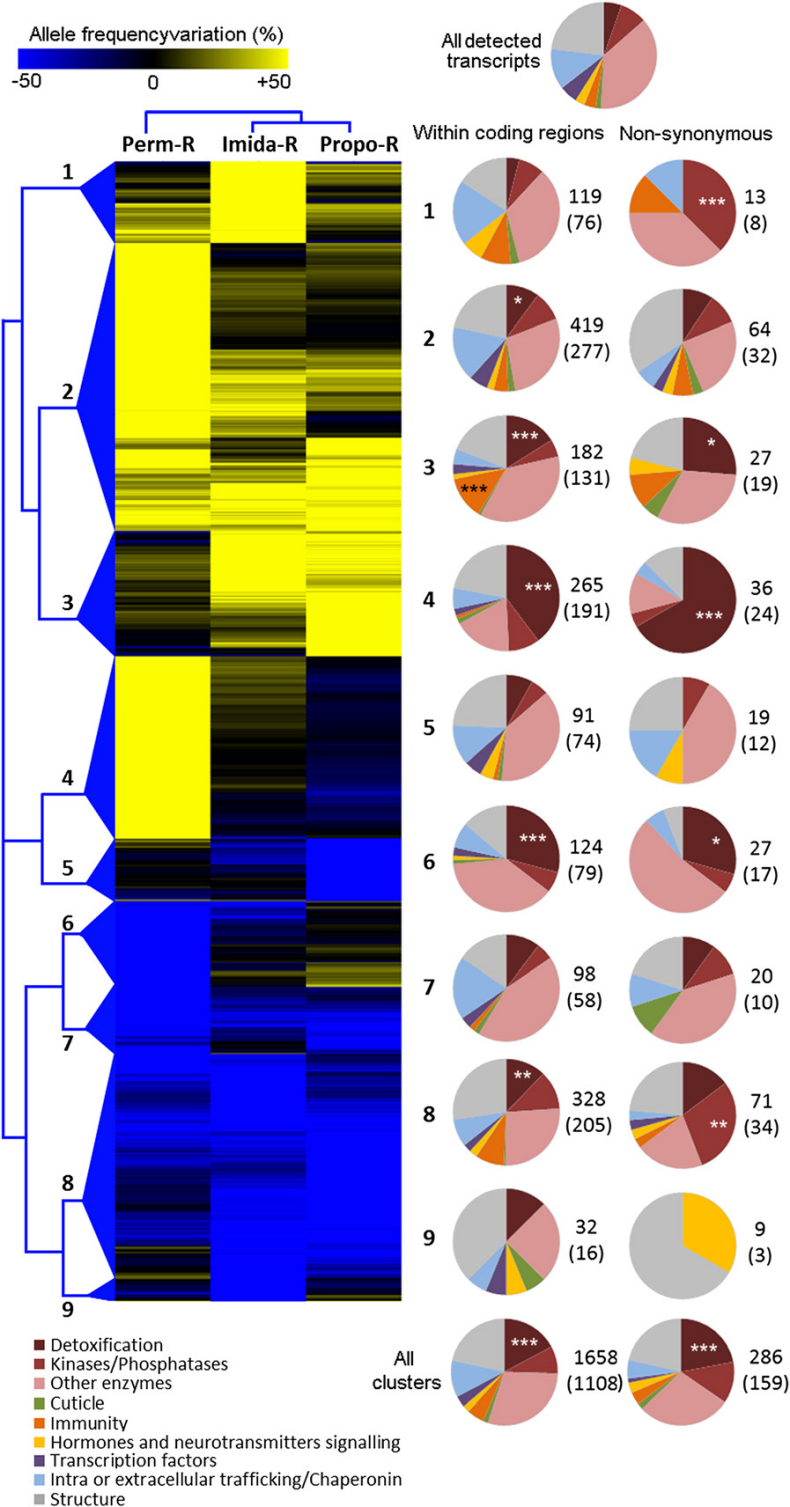
**Clustering of differential SNPs across strains.** The analysis was performed on all differential SNPs falling within coding regions. Clustering was based on Euclidean distance of allele frequency variations as compared to the susceptible strain and complete linkage algorithm. Pie charts describe biological functions affected within main clusters based on the number of differential SNPs affecting transcripts assigned to each function. Stars indicate biological functions significantly enriched compared to their representation among all detected transcripts (Fisher’s test adjusted P value: * ≤ 0.05, ** ≤ 0.01, *** ≤ 0.001). For each cluster, the total number of differential SNPs is indicated as plain text while those affecting transcripts of known function (used for pie charts) are shown within brackets.

More than 1650 differential SNPs were detected within coding regions (Figure 4 and Additional file 8: Table S5). Clustering analysis based on allele frequency variations between each insecticide-selected strain and the parental strain evidenced the differential response of each strain to insecticide selection. The Perm-R strain showed larger allele frequency variations as compared to other strains (median frequency variations of 50.2%; 29.8%; 27.9% for Perm-R, Imida-R and Propo-R respectively). When comparing all detected transcripts with those affected by differential SNPs (Figure 4, all clusters), a significant enrichment of transcripts encoding detoxification enzymes was detected. Such enrichment was also observed when considering variations predicted as non-synonymous only. When considering each cluster independently, clusters 1 and 9, representing differential SNPs specific to the Imida-R strain, did not show any significant enrichment in any biological category. Clusters 2, 4 and 6 were composed of differential SNPs specific to the Perm-R strain and revealed a significant enrichment in transcripts encoding detoxification enzymes, with multiple cytochrome P450s affected. As observed for transcription level variations, several transcripts encoding hexamerins were affected by large polymorphism variations in the Perm-R strain (Additional file 8: Table S5). Clusters 3 and 8, representing differential SNPs found in the Imida-R and Propo-R strains, also showed an enrichment in detoxification enzymes, as well as an enrichment in transcripts involved in immune response.

### Gene ontology terms enrichment analyses

Gene Ontology (GO) term enrichment analyses were performed independently on transcripts significantly over- or under transcribed or affected by differential SNPs in their coding region (Additional file 9: Figure S4). These analyses confirmed the distinct response of each strain to insecticide selection. Among GO terms representing genes over transcribed in insecticide-selected strains, terms related to cuticle proteins were strongly enriched in the Imida-R strain. Terms related to oxygen transport or to hexamerins were enriched in both the Perm-R and Imida-R strains, together with those related to iron homeostasis and iron binding. Among GO terms represented by under transcribed genes, multiple terms related to immune response were enriched in insecticide-selected strains. When considering transcripts affected by differential SNPs, no GO term was enriched in the Imida-R strain while only ‘dehydrogenase activity’ was enriched in the Propo-R strain. In contrast, the Perm-R strain revealed an enrichment of all terms related to cytochrome P450s such as ‘monooxygenases activity', ‘electron carrier activity', ‘tetrapyrrole binding', ‘heme binding’ and ‘iron ion binding’. Terms related to hexamerins were also enriched in Perm-R together with terms related to various enzyme families.

### Focus on transcripts potentially involved in insecticide detoxification

**Figure 5:**
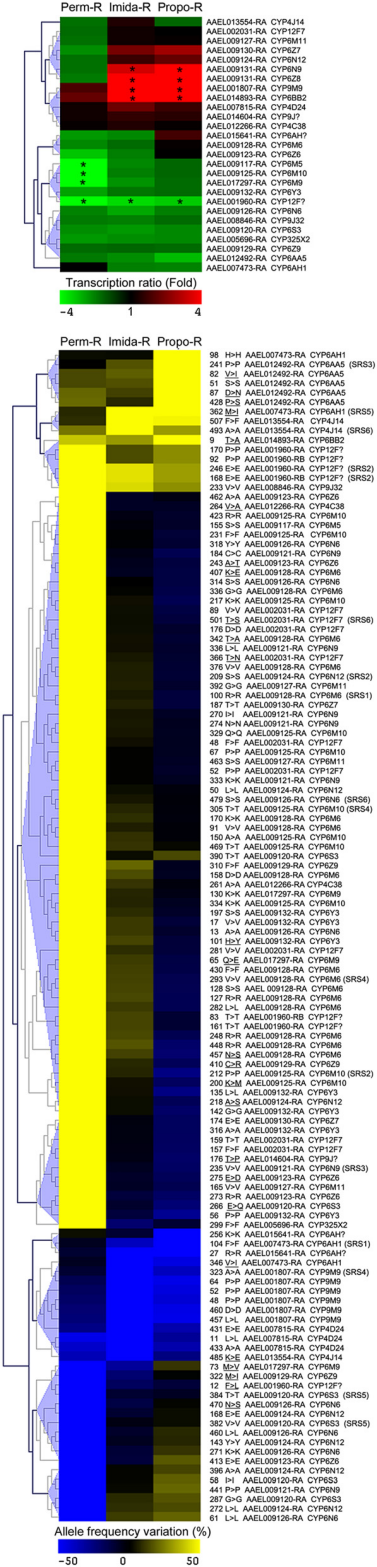
**Focus on detoxification: cytochrome P450s.** Clustering analyses of transcription level variations (upper panel) and differential SNPs (lower panel) affecting cytochrome P450 monooxygenases (CYP genes). Clustering was performed on all transcripts showing significant transcription level variations or differential SNPs within their coding region in any strain. The green-red color scale indicates transcription level variations as compared to the susceptible strain. Stars indicate a significant differential transcription. Blue-yellow color scale indicates allele frequency variations as compared to the susceptible strain. Amino acid position, amino-acid change, transcript number and gene names are indicated. Non-synonymous variations according to genome annotation are underlined. SNPs falling within P450 Substrate Recognition Sites (SRS) are indicated. Names ending with a question mark indicate genes with ambiguous gene name (subfamily indicated).

Focusing on cytochrome P450 monooxygenases (CYP genes, Figure 5) revealed that few *CYPs* showed significant transcription level variations, with none being over transcribed in the Perm-R strain and only four *CYPs* (*CYP6BB2*, *CYP9M9*, *CYP6N9* and *CYP6Z8*) being over transcribed in the Imida-R and Propo-R strains. Four *CYPs* were under transcribed in the Perm-R strain (*CYP6M5*, *CYP6M10*, *CYP6M9* and a *CYP12F*). This *CYP12F* was also under transcribed in the two other strains. When considering differential SNPs, the Perm-R strain carried a much higher number of differential SNPs affecting P450s as compared to other strains. The 19 *CYP* genes specifically affected by permethrin selection included 13 *CYP6s* belonging to a dense P450 cluster located on supercontig 1.371 composed of six *CYP6Ns*, five *CYP6Ms*, four *CYP6Zs*, *CYP6S3* and *CYP6Y3* (Additional file 10: Figure S5). In contrast, only 5 and 4 *CYPs* were affected by differential SNPs in the Imida-R and Propo-R strains respectively. Several *CYPs* were affected by variations predicted as non-synonymous (26 variations affecting 16 genes) while 17 variations affecting 14 genes where located within substrate recognition site regions (SRS), potentially affecting the active site of these P450s.

**Figure 6:**
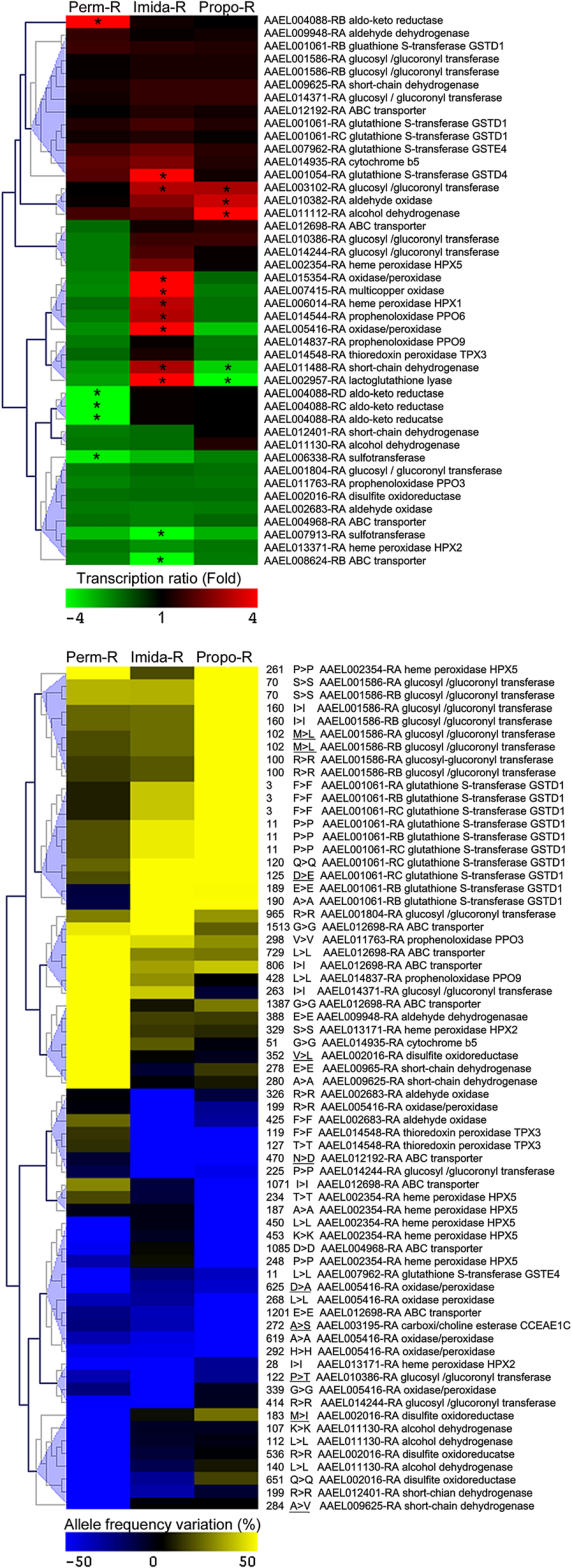
**Focus on detoxification: Other enzymes and transporters.** Clustering analyses of transcription level variations (upper panel) and SNPs (lower panel) affecting enzymes and transporters potentially involved in insecticide detoxification. Only transcripts or differential SNPs falling within coding regions are shown. Green-red color scale indicates transcription level variations as compared to the susceptible strain. Stars indicate a significant differential transcription. Blue-yellow color scale indicates allele frequency difference as compared to the susceptible strain. Amino acid position, amino-acid change, transcript number and gene names are indicated. Non-synonymous variations according to genome annotation are underlined.

When considering other transcripts potentially involved in resistance (Figure 6), the strong response of the Imida-R strain to insecticide selection through transcription level modifications was confirmed, with several detoxification enzymes being over expressed including the glutathione S-transferase *GSTD4*, one glycosyltransferase together with multiple oxidases, peroxidases and dehydrogenases. Several kinases were also specifically over-transcribed in the Imida-R strain. Fewer transcripts were affected in the Perm-R and Propo-R strains, including one aldo-keto reductase, one glycosyltransferase, one aldehyde oxidase and one alcohol dehydrogenase. As opposed to *CYP* genes, differential SNPs affecting other detoxification genes were well-balanced between strains with 66 variations affecting 26 distinct genes. Among them, ten variations were predicted as non-synonymous. In the Perm-R strain, ten genes were specifically affected, including two short-chain dehydrogenases, two heme peroxidases, two prophenoloxydases, one alcohol dehydrogenase, the glutathione S-transferase *GSTE4* and one ABC transporter. Ten variations affecting seven genes including the *GSTD1*, three glycosyltransferases, one aldehyde oxidase, one ABC transporter and one oxidase/peroxidase were specific to the Imida-R strain. Finally, 24 variations affecting seven genes including the *GSTD1*, one glycosyltransferase, one heme peroxidase, one carboxylesterase, two ABC transporters and one oxidase/peroxidase were specific to the Propo-R strain.

## Discussion

Characterizing resistance mechanisms is essential for improving resistance management. Although target site modifications play a major role in resistance [4,5,28,29], other mechanisms such as insecticide biodegradation, altered transport, sequestration and modification of the insect cuticle also account for a significant part of resistance [6,18,30,31]. However, the intricacy of these mechanisms makes challenging the identification of candidate genes for functional validation [21]. Gene expression microarrays are mostly used for identifying genes differentially transcribed in resistant populations but suffer from technical biases [21,32,33]. In contrast RNA-seq generates transcription data with a higher resolution, better dynamic range and lower technical variation [27]. In addition, RNA-seq produces polymorphism data and useful information to re-annotate gene models [25,26]. Despite recent studies pointing out the role of polymorphism variations in insecticide resistance [22-24], such aspect has never been investigated at the transcriptome level in mosquitoes. Indeed, the only study using RNA-seq to investigate insecticide resistance in mosquitoes did not consider polymorphism variations [34]. In this regard, the present study represents the first attempt to use RNA-seq for examining concomitantly quantitative and qualitative transcriptome changes associated with resistance to different insecticides in mosquitoes.

### Insecticide selection and resistance levels

After 10 generations of selection, bioassays revealed a constitutive increased resistance of each selected strain to its respective insecticide compared to the susceptible parental strain. Although resistance levels were low as compared to what can be observed *in natura*, they were significant regarding the few generations of selection, the full susceptibility of the parental strain and the absence of target-site resistance alleles. The rapid rise of resistance suggests that alleles conferring a better fitness in presence of insecticides are already present in susceptible populations and can be promptly selected under a constant selection pressure. Although laboratory selection does not fully mimic adaptive processes occurring *in natura* (e.g. lower population sizes, different environment, no introduction of resistant alleles by migration …), selecting all resistant strains from a single susceptible strain allowed minimizing variations related to different genetic backgrounds. Using a fully susceptible parental strain also allowed focusing on non-target site resistance mechanisms (no target-site mutations detected in parental or selected strains).

### RNA-seq as a tool for studying insecticide resistance in mosquitoes

A total of 270 million cDNA reads were sequenced across all samples and 80% of them were successfully mapped to *Ae. aegypti* reference genome. Such mapping efficiency appeared acceptable considering polymorphism variations occurring between the reference genome (Liverpool strain) and the parental strain used in our study (Bora-Bora strain). The high correlation of expression data obtained from cDNA library replicates confirmed the robustness of cDNA library construction and sequencing procedures. By applying high-stringency sequence quality and transcript coverage filtering, high fidelity transcription data were recovered for more than 13000 transcripts. Such detection level was comparable to those obtained with DNA microarrays at the same life stage [35,36]. More than 2500 differential SNPs linked to insecticide selection were identified. Although our experimental design did not control for stochastic effects (genetic drift) and the presence of false positives is likely, transcripts affected by these differential SNPs represent strong candidates for further functional validation studies. Little overlap was found between transcripts differentially transcribed in selected strains and those affected by differential SNPs. This was expected as RNA-seq data are restricted to transcripts and did not cover regulatory regions often located outside transcript boundaries. Challenging reads distribution with genome annotation identified > 3000 transcripts incorrectly annotated with most of them showing wrong UTR boundaries or modification of exon/intron structure. In addition, more than 500 lonely genomic regions showing high transcription signals and realistic exon/intron structures were identified. Further analyses are now required for assigning them to new exons of known transcripts, novel transcripts, pseudogenes or non-coding RNAs.

### Transcriptome changes associated with insecticide selection

Overall, our study revealed diverse response patterns depending on the insecticide used for selection. The Imida-R strain selected with the neonicotinoid imidacloprid showed the highest resistance level together with the strongest differential transcription response with numerous genes being over transcribed. In contrast, the Perm-R strain selected with the pyrethroid permethrin revealed a moderate differential transcription response but a lot of polymorphism variations following insecticide selection. The Propo-R strain selected with the carbamate propoxur showed an intermediate pattern. These dissimilar quantitative and qualitative transcriptome changes may reflect different adaptive strategies driven by costs and benefits associated with resistance mechanisms to each insecticide.

#### ***Insecticide resistance and immunity***

Multiple transcripts related to immune response were affected by insecticide selection with several of them under transcribed in all resistant strains (cecropins, defensins, lectins, …) and others affected by differential SNPs (defensins, clip-domain proteases, spatzle proteins…). Mosquito humoral response is involved in their capacity to host and transmit viruses [37] or parasites [38]. As mentioned in Alout et al. [39], our results support an association between insecticide resistance and the capacity of mosquitoes to host and transmit pathogens, which may affect the control of mosquito-borne diseases [40].

#### ***Altered insecticide transport and sequestration***

Multiple transcripts encoding cuticular proteins were over transcribed in the Imida-R strain while most of them were under transcribed in the Perm-R strain and not affected in the Propo-R strain. Such strong effect in the Imida-R strain was not associated to changes in the polymorphism of these transcripts, suggesting that insecticide selection is affecting their regulation rather than their conformation. Cuticle plays a crucial role in protecting insects from their environment. The vast majority of chemical insecticides are active by contact and changes in cuticle thickness or conformation have been suggested to contribute to resistance in mosquitoes [31,41,42]. Our data support previous studies suggesting a significant role of cuticle in the adaptation to neonicotinoid insecticides [36,43,44]. The role of cuticle in response to imidacloprid selection was supported by the specific over transcription of the multi-copper oxidase AAEL007415 in the Imida-R strain as its *An. gambiae* orthologue is involved in cuticle and egg shell tanning [45,46]. Numerous protein families are involved in cuticle biosynthesis and homeostasis including enzymes, transporters and transcription factors and further studies are now required for pinpointing those controlling cuticular resistance in mosquitoes.

Several kinases were specifically over-transcribed in the Imida-R strain. Kinases are involved in multiple regulatory mechanisms and their involvement in the response of insects to insecticides is likely. Indeed, recent studies showed that the phosphorylation state of acetylcholine nicotinic receptors can modulate the efficacy of neonicotinoid insecticides [47-49].

Five transcripts encoding hexamerins were strongly over transcribed in the Perm-R strain and in a lesser extend in other resistant strains. These transcripts, located on different supercontigs, were also affected by differential SNPs suggesting a selection imprint on these proteins. Insect hexamerins may be involved in hormone transport, energy and amino acid storage, cuticle biosynthesis and immune defense [50,51]. Hexamerins of the lepidopteran *Heliothis zea* have been shown to bind insecticides, suggesting a direct role in resistance by sequestration or altered transport [52]. Deciphering if hexamerins are impacted by insecticide selection because of their ability to bind insecticides, their interaction with cuticle homeostasis or their role in resources re-allocation associated to fitness costs remains unclear.

Finally, ATP-binding cassette transporters (ABC transporters) can play a role in adaptation to xenobiotics [53] and have been associated to insecticide resistance in mosquitoes [30,54]. In our study, the response of ABC transporters to insecticide selection was marginal. Indeed, only one ABC transporter (AAEL008624) was found differentially transcribed in response to insecticide selection and this gene was down regulated in all resistant strains. Four others were affected by differential SNPs but their allele frequency variations in resistance strains were low.

#### ***Insecticide biodegradation***

Detoxification enzymes play a major role in insecticide resistance [4,6,13,14,18,55]. As expected, these enzymes were well represented in our data set but showed distinct patterns depending on the nature of the insecticide used for selection.

Response to selection with the neonicotinoid imidacloprid (Imida-R strain) was characterized by the over transcription of multiple P450s, oxidases, transferases and one alcohol dehydrogenase, supporting the involvement of multiple enzymes in imidacloprid biodegradation pathways. Among them, the P450 CYP6BB2 was recently pointed out as a solid candidate for imidacloprid metabolism based on gene expression data and substrate binding predictions [36]. The aldehyde oxidase AAEL002683 was over transcribed and affected by polymorphism variations in the Imida-R strain. A recent study confirmed that aldehyde oxidases can contribute to neonicotinoid metabolism through nitro-reduction [56].

Several P450s over transcribed in the Imida-R strain were also found over transcribed in response to propoxur selection. Cross resistance between these two insecticides was identified [36] and the potency of particular P450s to confer resistance to multiple insecticides has been shown. For example, *An. gambiae* CYP6Z1 metabolizes the organochlorine DDT and the carbamate carbaryl [22] while CYP6M2 metabolizes both DDT and pyrethroids [57,58].

Metabolic resistance of mosquitoes to pyrethroids has been mainly associated with an over expression of P450s able to metabolize them. Among the multiple candidates identified by microarray screenings, *An. gambiae* CYP6M2 and CYP6P3, *An. funestus* CYP6P9, *Ae. aegypti* CYP9J32, CYP9J24, CYP9J28, and *Cx quinquefasciatus* CYP6M10 and CYP4H24 have been validated as pyrethroid metabolizers [review in 18]. CYP6Zs and CYP6Ms have also been associated with pyrethroid resistance by QTL in *An. funestus* where resistance mainly relies on metabolic mechanisms [59]. Recently, the central role of mosquito CYP6Zs in pyrethroid degradation pathway was revealed [55]. In the present study few P450s were found over transcribed in the Perm-R strain. Among them, CYP6BB2 was also found strongly over transcribed in pyrethroid resistant populations from Cuba and Cayman islands [30]. Unexpectedly, known *Ae. aegypti* pyrethroid metabolizers or paralogs of those validated from other mosquito species were not found over transcribed in the Perm-R strain. However, a strong selection imprint was detected in the Perm-R strain in a P450 cluster in supercontig 1.371containing strong candidates CYP6Ms, CYP6Ns and CYP6Zs, suggesting that the selection of particular P450 variants can contribute to pyrethroid resistance. In mammals, it is well known that P450 variants can display different substrate specificity [60-64]. To date, such variations have been neglected in mosquitoes with only few studies pointing out P450 alleles associated with insecticide resistance [22,23]. Finally, the strong selection imprint observed in these P450s might explain their unexpected under transcription in the Perm-R strain. Indeed, the apparent under expression of transcripts affected by differential SNPs might be the consequence of a mapping bias due to a higher divergence from the reference genome or an enrichment in low-expressed alleles [65,66]. Although further analyses are required for investigating the role of allele-specific expression in resistance, our data supports the selection of particular detoxification enzyme variants by insecticides.

## Conclusions

The present study primarily aimed at assessing the usefulness of RNA-seq to investigate insecticide resistance mechanisms in mosquitoes. Results confirmed that this technique produces high-quality gene expression data together with solid polymorphism data. Distinct responses to selection with insecticides from different chemical families were observed with a balance between gene expression and polymorphism variations. Polymorphism variations of P450 enzymes were strongly linked to pyrethroid selection. Although additional analyses are required to validate variants linked to resistance, such finding highlights the necessity to consider both gene expression and polymorphism variations for identifying candidate genes potentially involved in insecticide resistance. As sequencing costs are decreasing and new sequencing strategies and bioinformatics pipelines are developed, obtaining gene expression and polymorphism data from the same samples using high-throughput sequencing should now be considered as a valuable alternative to microarrays.

## Methods

### Mosquito selection with insecticides and bioassays

The mosquito *Ae. aegypti,* was used in the present study. Mosquitoes were reared in standard insectary conditions (26°C, 14 h/10 h light/dark, 80% relative humidity) in tap water (larvae) and net cages (adults). Larvae and adults were fed with hay pellets and papers impregnated with honey respectively. Blood feeding of adult females was performed on mice. Mice were maintained in an animal house agreed by French Ministry of animal welfare (n° B 38 421 10 001) and used in accordance to EU laws and the relevant ethic committee recommendations (ComEth Grenoble - C2EA - 12). The laboratory strain Bora-Bora, originating from French Polynesia and fully susceptible to insecticides, was used as a parental strain to select three independent strains with the pyrethroid insecticide permethrin (Perm-R strain), the neonicotinoid insecticide imidacloprid (Imida-R strain) and the carbamate insecticide propoxur (Propo-R strain). Both pyrethroid and carbamate insecticides are heavily used against mosquitoes and resistance to these insecticides has been reported worldwide. Neonicotinoids are marginaly used against mosquitoes but represent one possible alternative when resistance to other insecticide threatens the efficacy of mosquito control [36]. Selection was performed by exposing early 4th-stage larvae for 24 h to a lethal dose of each insecticide. For each strain, the dose of insecticide was adjusted at each generation (4 to 6 μg/L permethrin, 500 to 1000 μg/L imidacloprid and 500 to 800 μg/L propoxur) in order to reach 60-80% larval mortality after 24 h exposure. Surviving larvae were transferred in clean tap water, fed with standard larval food and allowed to emerge. Adults were allowed to reproduce for 4 days and blood-fed to obtain eggs for the next generation. In order to limit bottleneck effects, each generation was seeded with more than 6000 individuals. Selection process was carried out in parallel for all strains during ten generations. During this process, the susceptible parental strain was maintained in similar condition without insecticide selection. Bioassays and molecular work were performed on early 4th-stage larvae of the 11th generation (G_11_ larvae) bred in standard conditions and not exposed to insecticides.

To assess the constitutive resistance level of each selected strain, larval bioassays were conducted with permethrin, imidacloprid and propoxur comparatively to the susceptible parental strain. Five doses of each insecticide and four replicates of 25 larvae per dose were used. Doses of permethrin (1.5 to 6.5 μg/L), imidacloprid (150 to 2200 μg/L) and propoxur (100 to 2000 μg/L) were chosen in order to cover the whole mortality range after 24 h exposure. Lethal concentrations corresponding to 50% mortality (LC_50_) and their 95% confident intervals (CI_95%_) were then calculated with a probit approach for each strain using XL-Stat (Addinsoft, Paris, France). Resistance ratios (RR_50_ based on LC_50_ values) were calculated by comparison to the susceptible parental strain.

### RNA extraction and cDNA libraries preparation

For each strain, total RNA was extracted from G_11_ larvae obtained from three independent egg batches (three biological replicates per strain). Each biological replicate consisted of 180 larvae reared in 200 mL tap water in standardized insectary conditions. For each biological replicate, total RNA was extracted from 60 4th-stage larvae using the RNAqueous-4PCR kit (Applied Biosystems/Ambion, Austin, TX, USA), according to the manufacturer’s instructions. Total RNA quality and quantity were assessed with a Nanodrop ND1000 (Thermo Scientific, USA) and a 2100 Bioanalyzer (Agilent, USA). After extraction, total RNAs from each biological replicate were pooled in equal quantity to obtain a total RNA mixture representative of 180 individuals. Total RNA pools from each strain were then used for preparing cDNA libraries using the mRNA-Seq Sample Prep Kit (Part 1004898 Rev D, Illumina, USA). Two replicates of cDNA libraries were prepared for each strain as follows. Briefly, mRNAs were purified using poly-T beads and chemically fragmented. These fragmented mRNAs were reverse-transcribed using Superscript II (Invitrogen) at 42°C for 50 min. Double-stranded cDNAs were then synthesized and mRNAs removed using DNA polymerase I and RNase H at 16°C for 2.5 hours. Double-stranded cDNAs were purified using QIAquick PCR Purification Kit (QIAGEN, Germany) and processed for end-repair and 3′ adenylation using Klenow polymerase. Sequencing adaptors were then ligated using DNA ligase. Adapter-ligated cDNA libraries were then purified on 2% agarose gel based on a size range of 200 ± 25 bp. Adapter-ligated cDNA libraries were then enriched by 15 PCR cycles with adaptor-specific primers using Phusion DNA polymerase (Finnzymes Oy). Enriched cDNA libraries were then purified using QIAquick PCR Purification Kit (QIAGEN, Germany) before quality control analysis on an Agilent 2100 Bioanalyzer.

### Sequencing, read mapping and genome re-annotation

Each double-stranded cDNA library was sequenced as single reads of 75 bp in a distinct flow cell lane with a Genome Analyzer II (Illumina) at the National Sequencing Center (Genoscope, Evry, France). Reads were then mapped to the *Ae.aegypti* genome sequence (Aaeg L1.2 gene set, http://vectorbase.org) using the Tophat algorithm with default parameters (http://tophat.cbcb.umd.edu, release 1.0.14) [67], leading to an average mapping rate of 80%. Bam files were then loaded into Genespring NGS version 12.5 (Agilent Technologies) for further analysis. Reads were then filtered based on the sequence quality (mean read quality ≥ 30 and with < 10 Ns) and mapping quality (alignment score ≥ 98), and non-primary multiply mapped reads were removed. The remaining reads were used for all subsequent analyses. Read coverage was computed and genome annotation was challenged based on read distribution and coverage. Default feature detection parameters were used (min exon length percentile 10; min intro length percentile 10; max intron length percentile 90; min exon RPKM percentile 50; min gene RPKM percentile 50; min gene length percentile 10; min exon RPKM with respect to host gene percentage 75; min number of reads in exon 10). Modified exon-intron structures and new putative transcripts were proposed based on read coverage, read splicing events and distance with respect to existing genes and transcripts. Reads per Kilobase exon Model per Million sequenced reads (RPKMs) were calculated for all known and putative new transcripts. Transcript RPKM values were used for assessing the variability between library replicates for each strain.

### Differential transcription analysis

In order to avoid estimating transcription ratios from low coverage transcripts, only the 13105 transcripts showing at least 0.5 RPKM per condition (~ 30 reads/kb) were considered for differential transcription analysis. Differential transcription of each transcript was then tested between insecticide-selected strains and the susceptible parental strain using an Audic-Claverie test (AC test) based on read counts [68] with a Benjamini and Hochberg’s multiple testing correction [69]. Transcripts showing an adjusted P value < 10^-15^ and a fold change > 3 in either direction were considered as differentially transcribed between insecticide-selected strains and the parental susceptible strain. RNA-seq transcription data from the Imida-R strain were compared with transcription data obtained from the same biological samples with the *Aedes aegypti* 15 K DNA microarray as previously described [36]. Only the 326 transcripts showing significant adjusted P values in both techniques were considered.

### Polymorphism detection and differential analysis

Detection of polymorphisms was performed based on the 174 million reads passing quality filters with the following parameters: confidence score threshold = 100, coverage > 20 reads, base quality cut off = 5, ignore locations within or next to homopolymer stretches > 10 nucleotides. Among all detected polymorphism variations, only SNP substitutions were considered for differential polymorphism analyses. SNP allele frequencies were then computed between each insecticide-selected strain and the susceptible strain. Allele frequencies were considered as differential between an insecticide-selected strain and the susceptible strain (hereafter named as differential SNPs) if the following conditions were fulfilled: *i)* Total read coverage at SNP position between both strains ≥ 50, *ii)* Strand bias at SNP position ≤ 50 for both strains and *iii)* Allele frequency difference between both strains > 50% in either direction. Potential genic effects of SNPs were computed by comparing SNP with reference genome annotation. Genic effects were defined as 5′UTR, Synonymous, Non-synonymous, Intronic, 3′UTR and Intergenic (*i.e.* close but not within gene boundaries).

### GO term enrichment analyses

Go term enrichment analyses were conducted on *i)* transcripts significantly over transcribed in insecticide-selected strains compared to the susceptible strain, *ii)* transcripts significantly under transcribed in insecticide-selected strains compared to the susceptible strain and *iii)* transcripts affected by differential SNPs in their coding sequence as compared to the susceptible strain. GO term enrichment analyses were performed separately on each transcript list versus all detected transcripts. Enrichment statistics were computed based on hypergeometric distribution and adjusted with Benjamini-Yekutieli’s multiple testing correction [70] which takes into account the dependency among GO terms. GO terms showing an adjusted P value ≤ 0.05 were considered as significantly enriched in insecticide-selected strains compared to the susceptible strain.

### Clustering analyses

All clustering analyses were performed with TM4 MEV version 4.3.02 [71] using Euclidean distance and complete linkage algorithm with optimization of gene and condition trees. Transcripts significantly differentially transcribed (see above for thresholds) in any insecticide-selected strain compared to the parental susceptible strain were clustered based on their TR (log_2_ ratios). Annotated transcripts represented in the main clusters were then assigned to nine biological categories based on their annotation (including GO terms). Categories were defined as follow: ‘Detoxification', ‘Kinases/Phosphatases', ‘Other enzymes', ‘Cuticle', ‘Immunity', ‘Hormones and neurotransmitters signaling', ‘Transcription factors', ‘Intra or extracellular trafficking/chaperonins’ and ‘Structure’. Enrichment of these categories compared to all detected transcripts was then computed using a one-side Fisher’s exact test followed by Benjamini and Hochberg multiple testing correction [69]. Categories showing a corrected P value < 0.05 were considered significantly enriched. A similar clustering analysis was performed with differential SNPs (see above for detailed criteria) falling in coding regions. Clustering was performed as described above using allele frequency variations (-100% to +100%) in each strain as compared to the susceptible strain. Differential SNPs were assigned to nine different biological categories (see above) in regard of the nature of the transcript affected. For each cluster, biological category enrichment was computed versus all detected transcripts as described above.

Clustering analyses were then focused on transcripts encoding genes potentially involved in insecticide detoxification pathways (detoxification *sensus lato*). Clustering of both transcription level variations and differential SNPs were performed as described above. Separate analyses were conducted for cytochrome P450 monoooxygenases (CYPs) and other detoxification transcripts.

## Competing interests

The authors declare that they have no competing interests.

## Authors’ contributions

JPD conceived and coordinated the study, prepared the samples, analyzed data and wrote the manuscript. FF performed statistical analyses and help to draft the manuscript. ACP contributed to data analysis and helped to draft the manuscript. RP, MAR and AB contributed to biological and molecular samples preparation. VN performed mapping of RNA-seq data. SR contributed to study design, participated in biological sample preparation, data analysis and manuscript preparation. All authors read and approved the final manuscript.

## Data access

RNA-seq sequence data have been deposited at EBI/SRA/ArrayExpress under the accession number E-MTAB-1635. Final gene expression data (RPKMs, Fold changes and adjusted P values) are available at http://vectorbase.org. Distribution of mapped reads across mosquito genome can visualized using the *‘configure this page’* and *‘RNAseq alignments’* options of the vectorbase *Ae. aegypti* genome browser (tracks ‘Bora-Bora control’, ‘Perm-R’, ‘Imida-R’ and ‘Propo-R’).

## Supplementary Material

Additional file 1: Table S1 Sequencing and mapping statistics. Click here for file

Additional file 2: Figure S1 RPKM correlation between cDNA library replicates. Each dot represents one transcript. Only transcripts showing more than 0.5 RPKM are shown. Click here for file

Additional file 3: Figure S2 Comparison of read coverage across strains. Read coverage are indicated for each strain as RPKM (log scale). Coverage distributions are compared for unmodified transcripts (top), re-annotated transcripts (bottom left), and new putative transcripts (bottom right). Click here for file

Additional file 4: Table S2 Overview of transcriptome re-annotation. Click here for file

Additional file 5: Table S3 Genomic location of all novel transcribed features. Click here for file

Additional file 6: Figure S3 Cross-validation of transcription levels between RNA-seq and microarrays. Comparison is based on transcription data obtained from the Imida-R strain versus susceptible strain. RNA-seq and microarray data were obtained from the same generation. Correlation was performed on the 326 transcripts showing a significant differential transcription level in both studies. Solid grey line represents an equal transcription ratio between both techniques. Grey dashed lines represent a two-fold variation. Click here for file

Additional file 7: Table S4 Transcripts differentially expressed after insecticide selection. For each transcript, transcription level (fold) compared to the susceptible strain, adjusted P value and RPKM are indicated. Clusters and biological functions as described in Figure 2 are indicated. Click here for file

Additional file 8: Table S5 Differential SNPs linked to insecticide selection. For each SNP, allele frequency variations in each strain compared to the susceptible strain are indicated together with the affected transcript, the cDNA position, the amino-acid position and the amino acid change as compared to the reference genome. Clusters and biological functions as described in Figure 4 are indicated. Click here for file

Additional file 9: Figure S4 GO terms enrichment analysis. Analysis was performed on all transcripts significantly differentially expressed or affected by differential SNPs in insecticide-selected strains as compared to the susceptible strain. GO terms associated to each transcript were extracted from Vectorbase. GO terms showing adjusted P values < 0.05 were considered significantly enriched. Click here for file

Additional file 10: Figure S5 Differential SNPs linked to permethrin selection in supercontig 1.371. Transcripts location and read coverage are indicated. Transcripts showing differential SNPs in the Perm-R strain as compared to the susceptible strain are shown in red. For each transcript, the number of differential SNPs and their predicted genic effects are indicated. Click here for file
