# Supplementary figures and images for: Comparative analysis of response to selection with three insecticides in the dengue mosquito Aedes aegypti using mRNA sequencing
Source: BMC Genomics. 2014 Mar 5;15:174. doi: 10.1186/1471-2164-15-174 (PMC4029067; doi:10.1186/1471-2164-15-174)

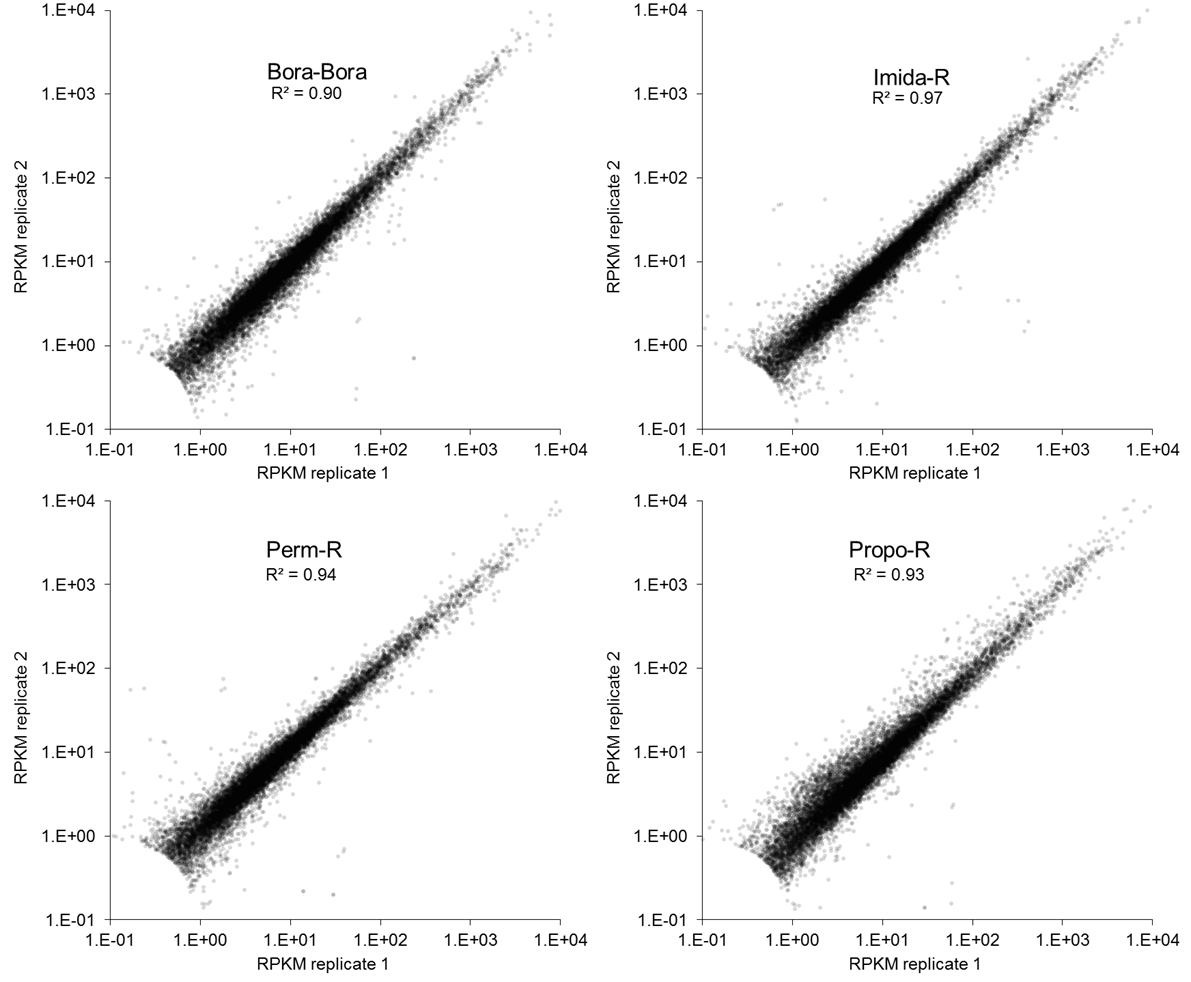

Supplement: Additional file 2: Figure S1 — RPKM correlation between cDNA library replicates. Each dot represents one transcript. Only transcripts showing more than 0.5 RPKM are shown. [file 1471-2164-15-174-S2.TIFF]

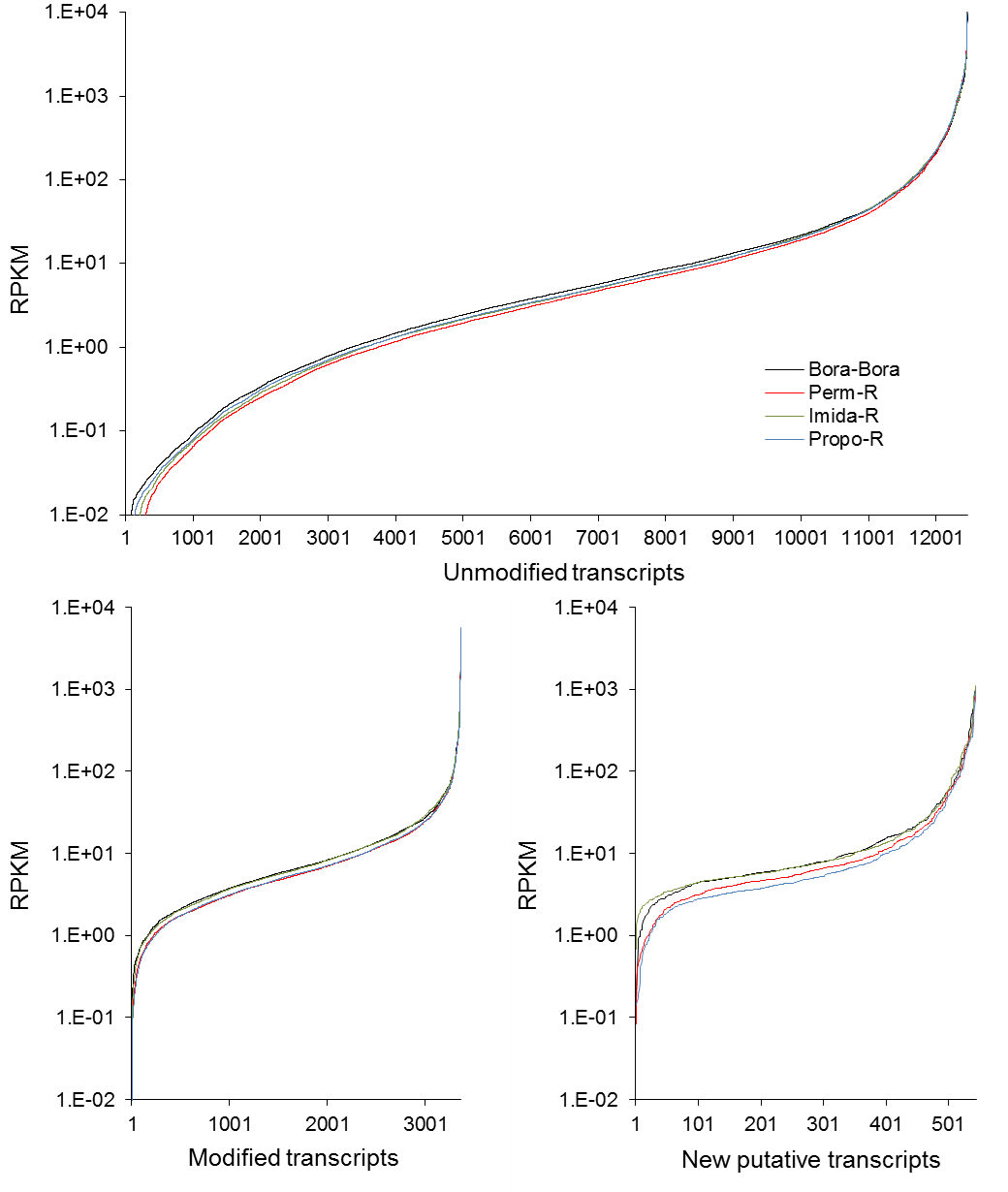

Supplement: Additional file 3: Figure S2 — Comparison of read coverage across strains. Read coverage are indicated for each strain as RPKM (log scale). Coverage distributions are compared for unmodified transcripts (top), re-annotated transcripts (bottom left), and new putative transcripts (bottom right). [file 1471-2164-15-174-S3.TIFF]

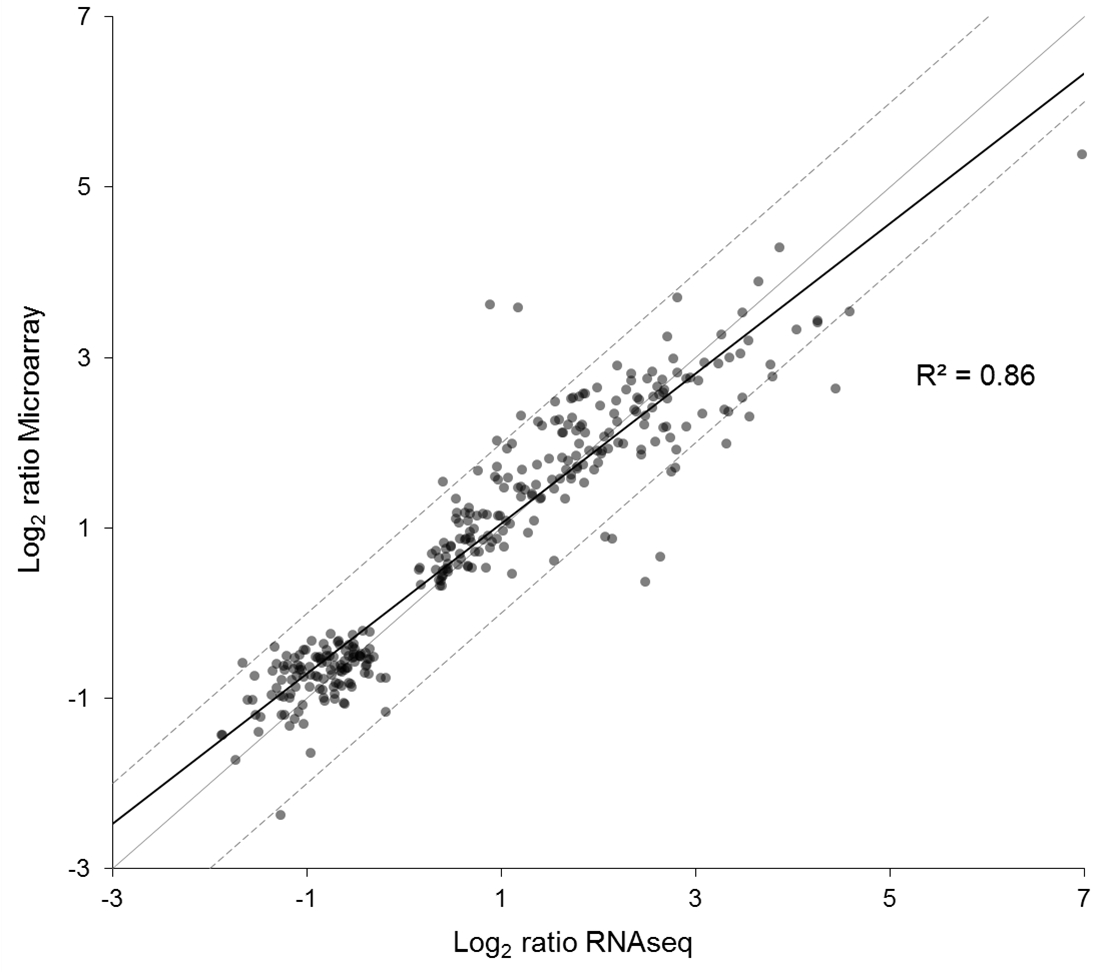

Supplement: Additional file 6: Figure S3 — Cross-validation of transcription levels between RNA-seq and microarrays. Comparison is based on transcription data obtained from the Imida-R strain versus susceptible strain. RNA-seq and microarray data were obtained from the same generation. Correlation was performed on the 326 transcripts showing a significant differential transcription level in both studies. Solid grey line represents an equal transcription ratio between both techniques. Grey dashed lines represent a two-fold variation. [file 1471-2164-15-174-S6.TIFF]

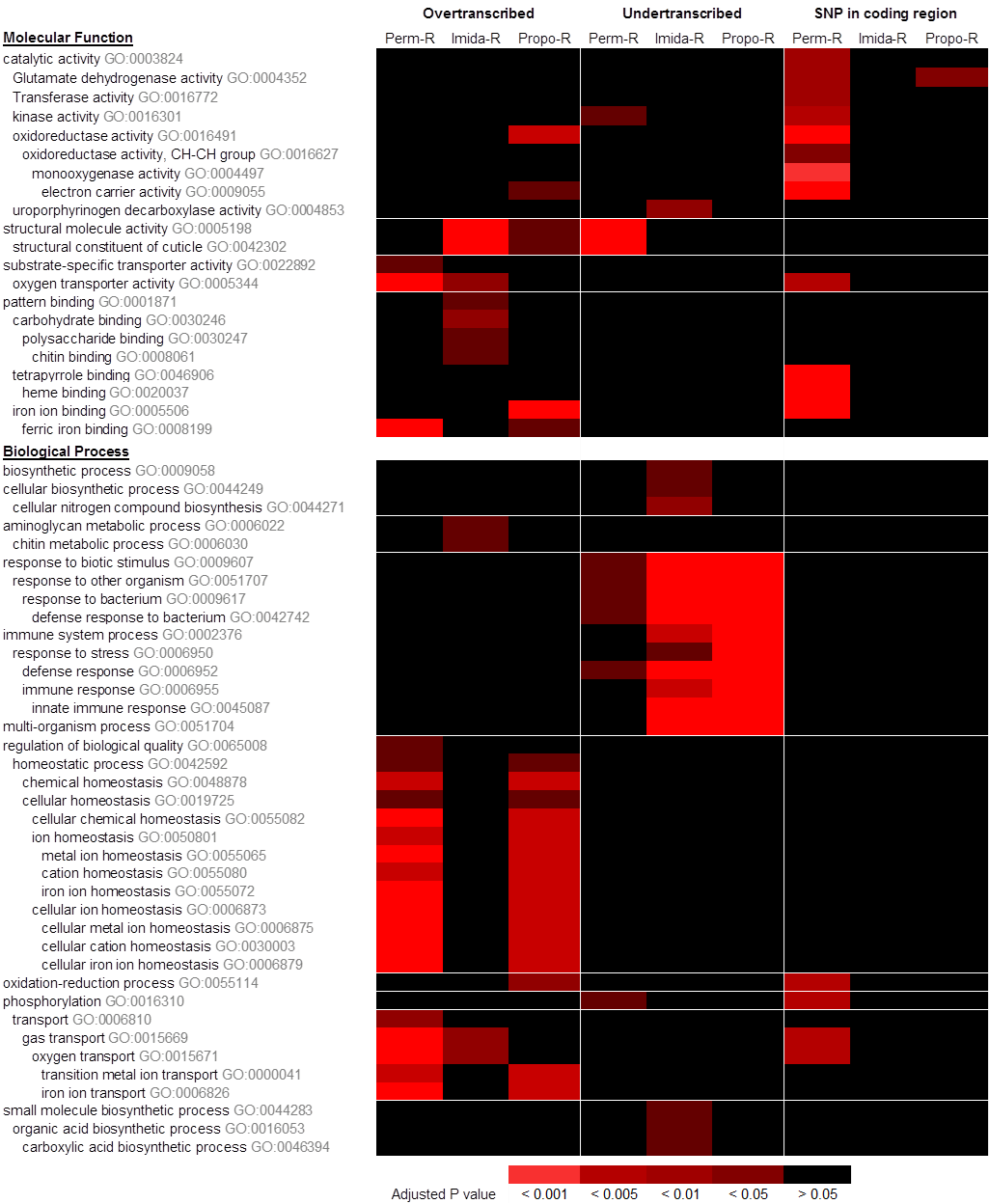

Supplement: Additional file 9: Figure S4 — GO terms enrichment analysis. Analysis was performed on all transcripts significantly differentially expressed or affected by differential SNPs in insecticide-selected strains as compared to the susceptible strain. GO terms associated to each transcript were extracted from Vectorbase. GO terms showing adjusted P values < 0.05 were considered significantly enriched. [file 1471-2164-15-174-S9.TIFF]

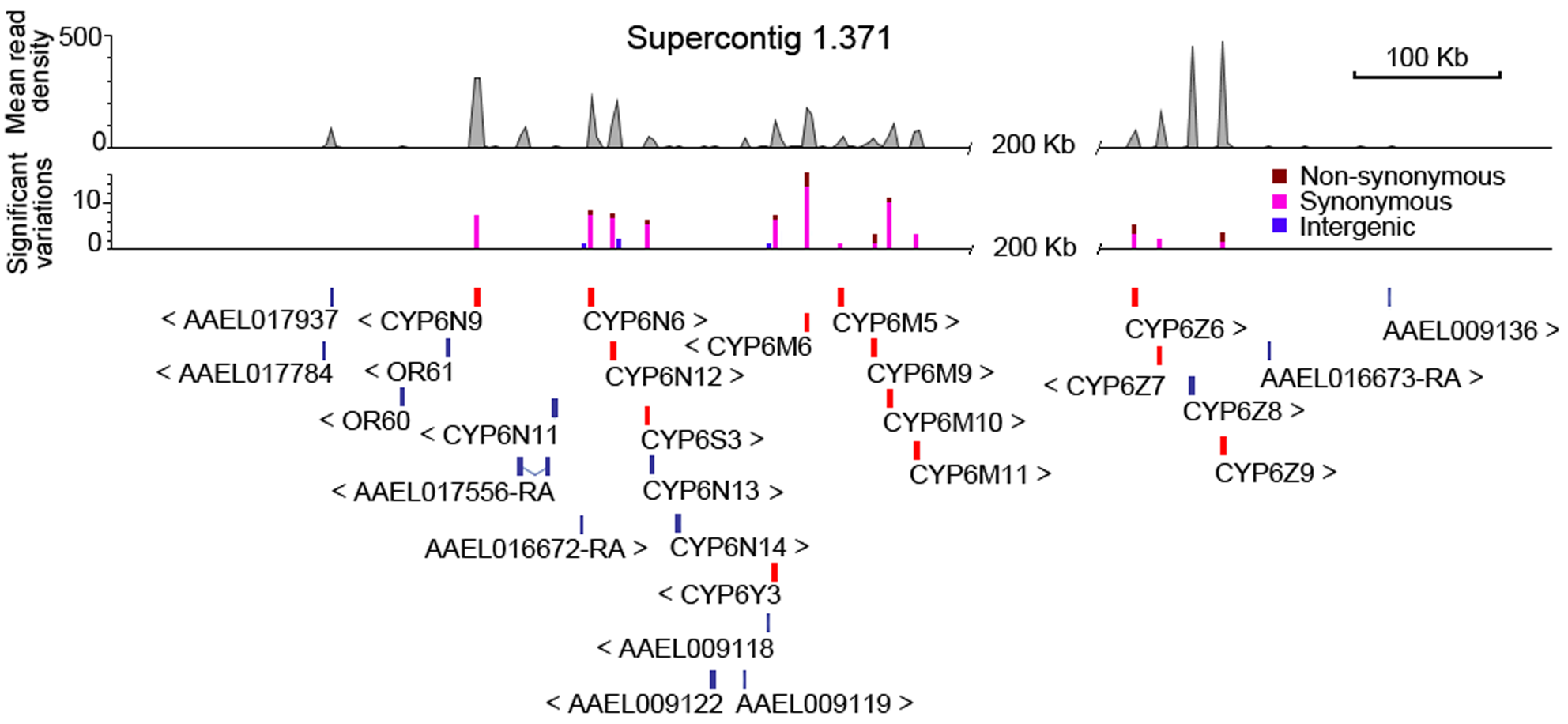

Supplement: Additional file 10: Figure S5 — Differential SNPs linked to permethrin selection in supercontig 1.371. Transcripts location and read coverage are indicated. Transcripts showing differential SNPs in the Perm-R strain as compared to the susceptible strain are shown in red. For each transcript, the number of differential SNPs and their predicted genic effects are indicated. [file 1471-2164-15-174-S10.TIFF]
